# Diarylethene-modified nucleotides for switching optical properties in DNA

**DOI:** 10.3762/bjoc.8.103

**Published:** 2012-06-20

**Authors:** Sebastian Barrois, Hans-Achim Wagenknecht

**Affiliations:** 1 Karlsruhe Institute of Technology (KIT), Institute for Organic Chemistry, Fritz-Haber-Weg 6, 76131 Karlsruhe, Germany

**Keywords:** absorption, cross coupling, molecular switches, oligonucleotide, palladium, photochromism

## Abstract

Diarylethenes were attached to the 5-position of 2’-deoxyuridine in order to yield three different photochromic nucleosides. All nucleosides were characterized with respect to their absorption and photochromic properties. Based on these results, the most promising photochromic DNA base modification was incorporated into representative oligonucleotides by using automated phosphoramidite chemistry. The switching of optical properties in DNA can be achieved selectively at 310 nm (forward) and 450 nm (backward); both wavelengths are outside the normal nucleic acid absorption range. Moreover, this nucleoside was proven to be photochemically stable and allows switching back and forth several times. These results open the way for the use of diarylethenes as photochromic compounds in DNA-based architectures.

## Introduction

The well-defined photoinduced switching of the optical and electronic properties of molecular components represents an increasingly significant goal for the development of new photoreactive nanostructured architectures. Among those architectures, nucleic acids were proven to be an important scaffold for the precise arrangement of all kinds of chromophores inside or along the double helix [1–4]. Nucleic acids are synthesized by building blocks; in this bottom-up approach artificial functionalities, such as photoswitches, can be introduced synthetically by providing the corresponding artificial DNA building block. Among the known and structurally diverse photochromic compounds [5–8], azobenzenes [9], spirobenzopyrans [10] and diarylethenes [11] represent the most promising candidates for introducing photoswitching functionality into biopolymers, and thereby for regulating biological activity [12–13]. Azobenzenes were designed and synthesized as artificial photoswitchable components in nucleic acids and are suitable for the control of a variety of different biological functions. The photoinduced cis–trans isomerization of azobenzene nucleosides can reversibly switch between the formation and dissociation of DNA duplexes [14–17], photoregulate DNA polymerase reaction [18], photocontrol DNA triplex formation [19], and drive photon-fueled DNA-based nanomachines [20–21].

**Scheme 1:**
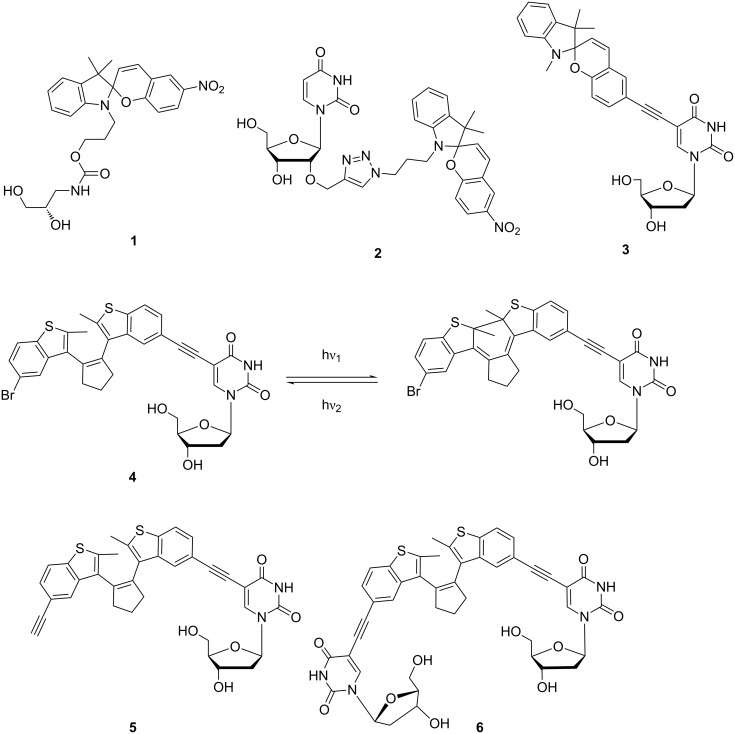
Spiropyran as DNA base surrogate **1**, DNA base modifications **2** and **3**, and diarylethene-modified nucleosides **4**–**6**. Photoswitching is representatively shown for nucleoside **4**.

Concerning the extent of structural change, the cis–trans isomerization of azobenzenes behaves much simpler than the ring opening of spiropyrans to merocyanines. In the latter case not only is a structural change observed but a large change in polarity is yielded additionally [10,22]. It is expected that the ring-closed spiropyran form of this molecular switch does not insert into the base stack due to its twisted structure, but the open merocyanine form could intercalate based on its planarity and polarity. This assumption was experimentally verified by synthetic spiropyrans as noncovalent DNA and RNA binders [23–25]. As expected, ground-state interactions between the noncovalently bound molecular switch and the DNA bases were detected in the case of the merocyanine form by CD spectroscopy, but not in the case of the spiropyran form. Attempts to attach spiropyrans covalently to DNA are rare [26–27] and include our recent approaches to incorporate synthetically the spiropyran chromophore [28], by either DNA building block **1** [29] or by DNA base modifications **2** and **3** [29–30], into oligonucleotides (Scheme 1). Although the spiropyran DNA building blocks **1** and **2** exhibit promising photochromic properties as nucleosides, it is not worth pursuing this synthetic direction further for the following reasons [30]: (i) It is reported that spiropyrans decompose in aqueous buffer solutions [31], and (according to our experience) the DNA environment does not efficiently prevent this decomposition. (ii) Moreover, we found out that the spiropyran chromophore in the DNA environment loses its photoswitching abilities [29].

The second alternative, diarylethenes, have been applied for switching the binding affinities of proteins and peptides [32] but are still rather unexplored with respect to nucleic acids. The advantage of this type of photoswitch is that back reaction (reopening of the central ring) requires light and cannot be achieved in a thermally induced way. Diarylethenes have been applied in a noncovalent chiroptical photoswitchable DNA complex [33] and combined with 7-deazaadenosine to obtain a nucleosidic diarylethene switch [34]. Recently, we reported, very preliminarily, the preparation and optical properties of nucleoside **4** [30]. Herein, we give a full account and present the detailed synthesis of diarylethene-modified nucleosides **4**, **5** and **6** (Scheme 1), and the characterization of their photochromic behavior. It turned out to be most promising to continue the DNA work with the modified and photochromic nucleoside **4**, which was, thus, incorporated into representative oligonucleotides by using automated phosphoramidite chemistry, and characterized with respect to its photochromism in DNA.

## Results and Discussion

### Design and synthesis of diarylethene-modified 2’-deoxyuridines **4**–**6**

The typical way to tether fluorophores to oligonucleotides is to use an alkyl chain linker between the chromophore and DNA base as the point of attachment. The purpose of this conformationally flexible tether is to overcome problems in the enzymatic activity by DNA polymerases in the context of primer-extension (PEX) or PCR studies [35–37]. However, the shortest possible linking of chromophores to oligonucleotides offers potentially interesting optical characteristics, among these being solvatochromism and red-shifted exciplex-type fluorescence [38]. Such absorption and fluorescence readouts are potentially suitable for DNA probing [39–41]. On the other hand, with respect to polymerase-assisted PEX and PCR experiments, an absolutely critical issue regarding the application of single C–C bonds, or ethinyl or phenylene linkers is the question of whether the canonical base-recognition complementarity is effected by the chromophore modification [42–43]. Due to the strongest reactivity the 5-position of pyrimidines (U/T and C) and the 8-position of purines (A and G) are preferred as chromophore modification sites. The assumption, that these points of attachment allow the chromophores to point into the major groove is only partially true, if at all. In particular, large and aromatic chromophores tend to insert into the base stack due to their hydrophobicity by changing the attached nucleoside from the natural anti- to the syn-conformation. The modification of the 8-position of purines clearly forces the nucleoside into the syn-conformation, which interferes with Watson–Crick base pairing. In the case of the pyrimidines the modification at position 5 should not significantly interfere with the preferred anti-conformation of the nucleosidic bond. Thus, the Watson–Crick base pairing of the corresponding modified oligonucleotides should be maintained. Over the past few years we have synthetically attached various chromophores, such as ethynylpyrenes [44–45], BODIPY [46], ethynyl nile red [47–48] and others, to 2’-deoxyuridines for electron transfer studies and for fluorescent probes. To gain greater insight into the counterbase selectivity, we performed PEX experiments with a representative set of chromophore-modified oligonucleotides and found that the DNA-polymerase-catalyzed nucleotide incorporation opposite to attached chromophores at the 5-position of uridine follows Watson–Crick selectivity [49]. In the meantime, DNA polymerases have been evolved that have an increased reactivity for C5-modified deoxyribonucleotides [50]. Hence, it looked most reasonable to attach diarylethenes as photochromic compounds to the 5-position of 2’-deoxyuridine. The nucleoside **4** bears the bromine group, which was introduced initially for synthetic reasons. In the second nucleoside **5** the bromine was replaced by an ethynyl group. The third nucleoside **6** carries two 2’-deoxyuridine moieties and should principally allow connection of the two DNA double helices by the diarylethene linker as a covalent bridge between.

**Scheme 2:**
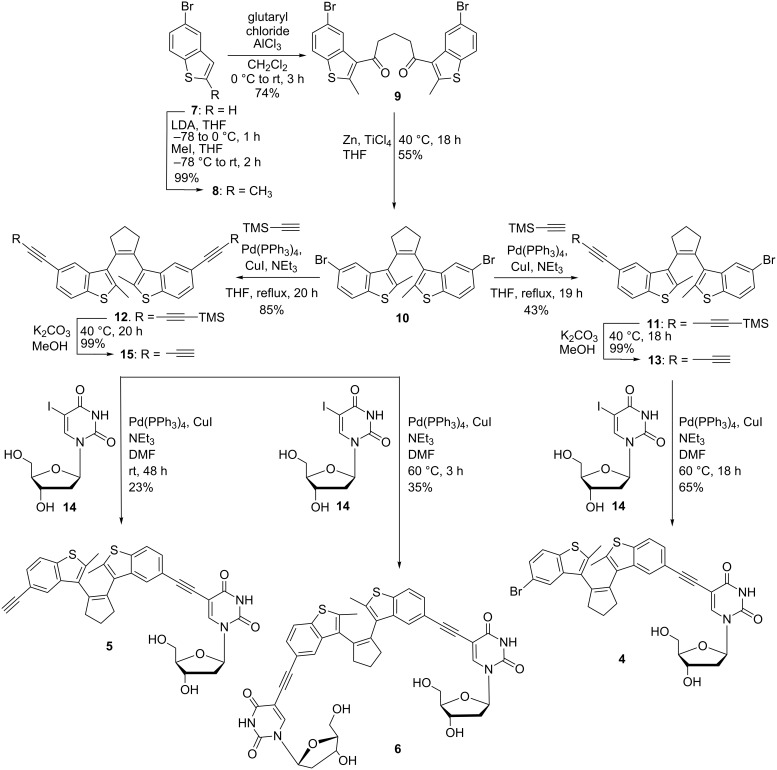
Synthesis of diarylethene-modified 2’-deoxyuridines **4** [30], **5** and **6**.

The preparation of all three nucleosides **4** [30], **5** and **6** (Scheme 2) is based on Sonogashira-type cross couplings as key steps [51] to attach the photoactive chromophore to the nucleoside. For the synthesis of the first nucleoside **4** (Scheme 2), commercially available 5-bromobenzothiazole (**7**) is deprotonated with LDA and methylated by methyl iodide in quantitative yield. The resulting 5-bromo-2-methylbenzothiazole (**8**) was converted in a double Friedel–Crafts-type acylation. Treatment with glutaryl chloride in the presence of AlCl_3_ connects two benzothiazoles **8** and provides **9** in 74% yield. The double McMurry-type reaction, which is performed with Zn and TiCl_4_, forms the central cyclopentane ring of diarylethene **10**. Subsequently, the ethynyl linker is attached to **10** by treatment with TMS-protected acetylene in a Pd-catalyzed cross-coupling reaction. Careful evaluation of this synthetic step revealed that the best yield (43%) of the mono-ethinylated product **11** is obtained with 2 equiv of TMS-acetylene in the reaction mixture. The competitively formed doubly ethinylated diarylethene **12** is, of course, easier to obtain. In the latter case, a strong excess of TMS-acetylene (10 equiv) helps to increase the yield of product **12** to 85%. The synthesis of the first diarylethene-modified nucleoside **4** then continues by cleavage of the TMS protecting group of **11** with K_2_CO_3_ in MeOH. Finally, the synthesis is concluded by the Sonogashira-type coupling of diarylethene **13** to 5-iodo-2’-deoxyuridine (**14**) in 65% yield.

For the second and third modified nucleosides **5** and **6**, the last two synthetic steps are similar to those for nucleoside **4**. Deprotection of **12** gave the doubly ethinylated diarylethene **15** in quantitative yield. The subsequent Sonogashira coupling with **14** at rt gave the mono-nucleosidic product **5** in 23% yield, and at 60 °C the di-nucleosidic product **6** in 35% yield.

### Photochromic properties of diarylethene-modified 2’-deoxyuridines **4**–**6**

**Figure 1:**
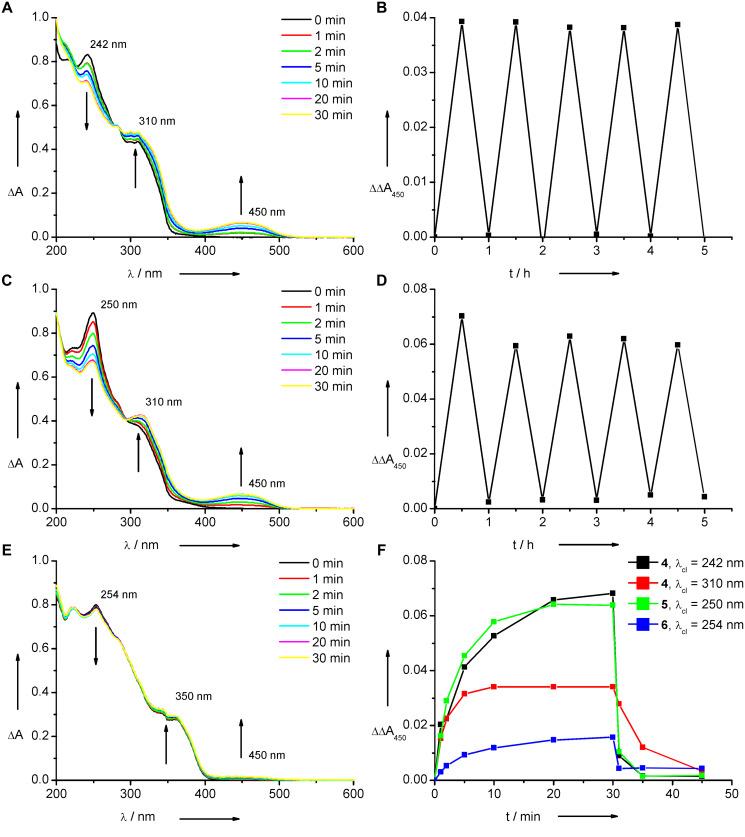
Photoswitching properties of nucleosides **4**–**6** (each 20 mM in MeCN, rt). Top: Irradiation of **4** at 242 nm (**A**, left) and irradiation of **4** alternating at 242 nm and 450 nm (**B**, right). Middle: Irradiation of **5** at 250 nm (**C**, left) and irradiation of **5** alternating at 250 nm and 450 nm (**D**, right). Bottom: Irradiation of **6** at 254 nm (**E**, left) and plots of kinetic traces of absorption changes at 450 nm of all nucleosides **4**–**6** irradiated at the corresponding wavelength until 30 min, then at 450 nm (**F**, right).

The photochromic properties of the modified nucleosides **4**–**6** were characterized by UV–vis absorption spectroscopy at rt. All irradiations for photoswitching of the nucleosides were performed by using a 75 W Xe arc lamp equipped with a monochromator for wavelength-selective excitation. The absorption of nucleoside **4** in the pure synthetic form (and presumably open form) shows absorption peaks in the UV range at 242 nm and 310 nm (Figure 1A). It is assumed that the band at 242 nm overlays with the absorption of the uracil moiety. Hence, the absorption side band at 310 nm should allow, at least in principal, the selective excitation of the switch, outside the nucleic acid absorption range. Accordingly, the photoswitching of **4** was probed at 242 and 310 nm. In fact, by irradiation at either wavelength the closed form of nucleoside **4** is formed with its characteristic band at 450 nm in the visible range. The color of the samples turns yellow. However, the differences in the absorption changes indicate that especially the contribution of the colored closed form of **4** in the photostationary state by excitation at 310 nm is less than at 242 nm (Figure 1F). Hence it can be concluded, that the band at 310 nm can be used indeed to excite selectively the diarylethene chromophore in nucleic acids, but the yield of the colored form is reduced. In all cases, the photostationary state was reached in 30 min since no additional changes were observed after that time. The absorption bands of the open and closed forms of **4** overlap in the UV range, which makes a detailed analysis of the photostationary state impossible. Irradiation of the sample at 450 nm reopens the diarylethene **4** within a few minutes. The absorption spectra of **4** during all irradiations show an isosbestic point at 275 nm. As expected, the thermally induced opening of the closed chromophore in nucleoside **4** was never observed. The diarylethene nucleoside **4** stays chemically and photochemically stable and can be switched several times to the closed form, and back to the opened form (Figure 1B).

At first glance, the photochromic properties of the second nucleoside (**5**) are very similar to those of **4** (Figure 1C). However, a careful look at the absorption changes performed at 250 nm reveals that the isosbestic point between the closed and the opened form of nucleoside **5** has moved bathochromically from 275 to 300 nm. Moreover, the closed form does not only show an increased absorption in the visible range at 450 nm but also a more significant contribution to the absorption in the UV range at 310 nm. As a result, photoswitching from the opened form of **5** to the closed form at 310 nm cannot be performed in an efficient way. This result is in contrast to that for the first diarylethene-modified nucleoside **4** described above and limits the applicability of nucleoside **5** significantly as a photochromic switch in nucleic acids. The diarylethene **5** can be switched several times back and forth; however, the photochemical stability is slightly reduced compared to nucleoside **4** (Figure 1D).

A rather dramatic change of photochromic properties is observed upon comparison of nucleosides **4** and **5** with the third one (**6**). The synthetically obtained and thereby purely closed form of diarylethene **6** shows absorption at 254 and 350 nm (Figure 1E). Irradiation at 250 nm indeed shows the formation of the colored form at 450 nm, but to a much smaller extent when compared with **4** or **5**. Obviously, the conjugation of the terminal acetylene group of nucleoside **5** to a second 2’-deoxyuridine in **6** affects its photochromism significantly and reduces its photoswitching abilities.

### Synthesis and photochromic properties of DNA modified with diarylethene **4**

**Scheme 3:**
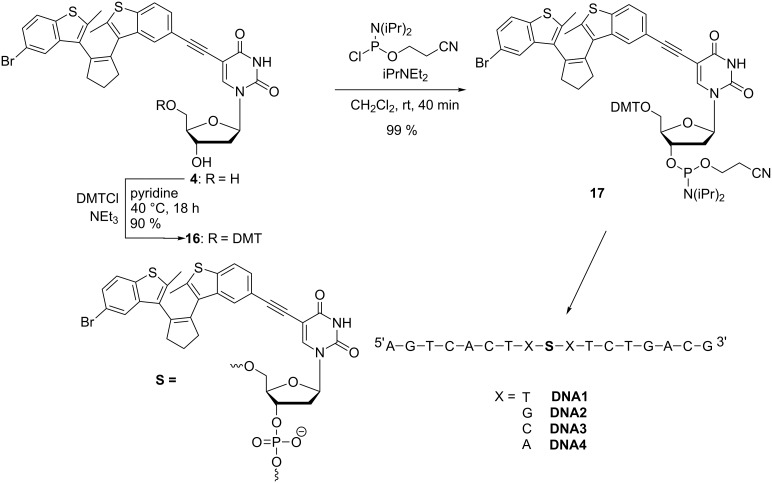
Synthesis of DNA building block **17** [30] and sequences of diarylethene-modified **DNA1**–**DNA4**.

As mentioned in the introduction, in a bottom-up approach nucleic acids can be modified with artificial functionalities by providing the corresponding synthetic building blocks. Based on the previously described results regarding the photochromic properties of nucleosides **4** [30], **5** and **6** it appeared to be most promising to incorporate synthetically the nucleoside **4** into oligonucleotides. It is the only diarylethene that allows selective excitation at 310 nm, outside of the absorption range of DNA. The preparation of the corresponding phosphoramidite **17** as an artificial DNA building block follows standard procedures (Scheme 3) [30]: Protection of the 5’-OH group of nucleoside **4** by DMT-chloride is followed by phosphitylation of the 3’-OH group of the intermediate **16** yielding phosphoramidite **17**. By automated DNA synthesis on a solid phase we prepared a set of four oligonucleotides in order to report preliminarily the optical properties of **4** in DNA. The sequences were identical except for the base pairs that surround the position of nucleoside **4**. The oligonucleotides were purified by semipreparative HPLC, identified by mass spectrometry and quantified by their extinction coefficient at 260 nm.

**Figure 2:**
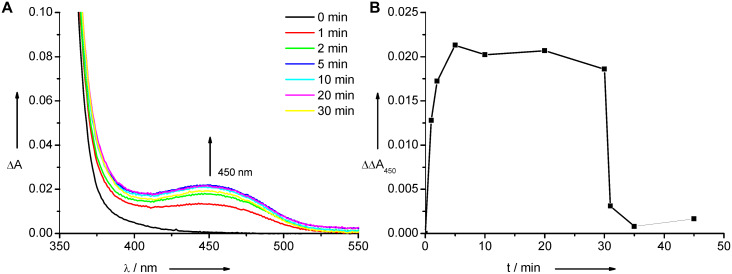
Irradiation of ds**DNA2** at 310 nm (**A**, left) and plot of kinetic trace of absorption changes at 450 nm of ds**DNA2** irradiated at 310 nm until 30 min, then 450 nm (**B**, right).

Irradiation of each of the four single-stranded oligonucleotides **DNA1**–**DNA4** at 310 nm yields the characteristic absorption of the closed forms as small and broad bands at ~450 nm, and irradiation at this wavelength drives the reaction back toward the open form of the switch (representatively shown for **DNA2** in Figure 2). The most remarkable result is that of **DNA2** bearing two guanines in the direct vicinity of the diarylethene modification. Guanine is known to interfere with a variety of photophysical properties (mainly fluorescence) in DNA. It is important to mention here that guanine does not influence the photochromic properties of the diarylethene switch **4** as a DNA modification. In fact, the photoswitching behavior of **4** is maintained in all four different DNA environments. The sequence independent photochromic behavior represents an important result for the future application of this type of switch in DNA-based nanostructures. Representatively, we performed switching experiments with the double strand (ds) of the most critical candidate (**DNA2**). The kinetic behavior of the opening and closing of nucleoside **4** cannot be directly compared to ds**DNA2** since the solvents in the two measurements are different. However, the photochromic switching is clearly observable in the absorption spectra (Figure 2A and 2B) and is very similar to that of the single strand. Double-stranded **DNA2**, with the diarylethene switch in the opened form, exhibits a melting temperature of 60.6 °C and shows a high destabilization according to the unmodified double strand (68.0 °C). After irradiation at 310 nm, the melting temperature decreases significantly to 56.8 °C. According to the noncovalently bound diarylethene derivative as chiroptical switch published by Feringa and coworkers [33], the closed form is able to intercalate whereas the open form of the switch binds differently. This could potentially explain the difference in melting temperature for ds**DNA2**, since the intercalated closed form of the diarylethene interferes with stacking to the neighboring bases by the two methyl groups pointing up and down from the chromophore.

## Conclusion

Three different diarylethene-modified 2‘-deoxyuridines **4**–**6** were prepared by using Sonogashira-type cross couplings as key steps. The photochromic properties of the nucleosides were evaluated and revealed that only nucleoside **4** allows selective excitation at 310 nm, outside the nucleic acid absorption window, to close the diarylethene chromophore. Switching back of the colored form of all three nucleosides **4**–**6** to the corresponding opened forms can be achieved by selective excitation at 450 nm. Moreover, nucleoside **4** was proven to be photochemically stable and, hence, allows switching back and forth several times. Nucleoside **4** was incorporated into oligonucleotides by automated DNA synthesis. It is remarkable that the photochromic properties of **4** are maintained for the corresponding base modification in oligonucleotides and show sequence-independent switching behavior. Our results open the way for the use of diarylethenes as photochromic compounds in DNA-based architectures and represent one important step further for the design and synthesis of photoreactive and self-assembled nanostructures and materials based on nucleic acids.

## Experimental

### Materials and methods

Chemicals were purchased from Sigma Aldrich, Fluka, ABCR, Alfa Aesar and Acros. Unmodified oligonucleotides were purchased from Metabion. TLC was performed on Merck silica gel 60 F_254_ coated aluminum foil. Flash chromatography was carried out with silica gel 60 from Aldrich (60–73 µm). Spectroscopic measurements and the melting points were recorded on a Varian Cary 100 spectrometer in quartz-glass cuvettes. Irradiation experiments were performed with a 75 W Xe arc lamp equipped with a monochromator. The synthetic procedures for compounds **4, 8**, **9**, **10**, **11**, **13**, **16** and **17** have been already described in [30].

**Compound 12: 10** (617 mg, 1.19 mmol), trimethylsilylacetylene (1.68 mL, 11.90 mmol), dry NEt_3_ (0.39 mL, 4.76 mmol), Pd(PPh_3_)_4_ (550 mg, 0.48 mmol) and CuI (91 mg, 0.48 mmol) were dissolved in dry THF (11 mL) under argon. The mixture was degassed and heated under reflux for 20 h. After cooling to rt, the solvent was removed under reduced pressure. The residue was dried in vacuo and purified by flash chromatography on silica gel (hexanes–toluene 100:1) to yield 559 mg **12** (85%) as a white solid. *R*_f_ 0.20 (hexanes); ^1^H NMR (300 MHz, CDCl_3_) δ 7.72 (s, 2H, H-Ar), 7.56 (d, *J* = 7.9 Hz, 2H, H-Ar), 7.30 (d, *J* = 6.9 Hz, 2H, H-Ar), 3.20–2.80 (m, 4H, CH_2_), 2.31 (p, *J* = 6.8 Hz, 2H, CH_2_), 1.99 (s, 6H, CH_3_), 0.30 (s, 18H, Si(CH_3_)_3_); ^13^C NMR (75 MHz, CDCl_3_) δ 139.3 (C_quart._), 138.7 (C_quart._), 137.5 (C_quart._), 137.1 (C_quart._), 130.2 (C_quart._), 127.1 (C_arom._), 126.1 (C_arom._), 121.9 (C_arom._), 118.7 (C_arom._), 106.0 (C≡C), 37.9 (CH_2_), 29.9 (C≡C), 24.3 (CH_2_), 15.4 (CH_3_), 0.3 (Si(CH_3_)_3_); EIMS (70 eV) *m*/*z* (%): 552.2 (8) [M]^+^; HRMS–ESI (*m*/*z*): [M]^+^ calcd for C_33_H_36_S_2_Si_2_, 552.1797; found, 552.1794.

**Compound 15: 12** (408 mg, 0.74 mmol) was dissolved in dry MeOH (15 mL), and K_2_CO_3_ (408 mg, 2.95 mmol) was added. The reaction mixture was stirred for 20 h at 40 °C. After cooling to rt, MeOH was removed under reduced pressure. The residue was dried in vacuo and purified by flash chromatography on silica gel (hexanes–toluene 10:1) yielding 299 mg **15** (99%) as a white solid. *R*_f_ 0.41 (hexanes–toluene 10:1); ^1^H NMR (400 MHz, CDCl_3_) δ 7.75 (s, 2H, H-Ar), 7.57 (s, 2H, H-Ar), 7.31 (s, 2H, H-Ar), 3.08 (s, 2H, C≡CH), 3.01–2.77 (m, 4H, CH_2_), 2.28 (p, *J* = 7.3 Hz, 2H, CH_2_), 1.96 (s, 6H, CH_3_); ^13^C NMR (101 MHz, CDCl_3_) δ 139.3 (C_quart._), 138.9 (C_quart._), 137.1 (C_quart._), 130.0 (C_quart._), 128.5 (C_quart._), 126.8 (C_arom._), 126.4 (C_arom._), 122.0 (C_arom._), 117.6 (C_arom._), 76.4 (C≡C), 37.9 (CH_2_), 29.9 (C≡C), 24.2 (CH_2_), 15.2 (CH_3_); EIMS (70 eV) *m*/*z* (%): 408.1 (48) [M]^+^.

**Compound 5: 15** (75 mg, 0.18 mmol), 5-iodo-2’-deoxyuridine (**14**, 65 mg, 0.18 mmol), dry NEt_3_ (30 µL, 0.37 mmol), Pd(PPh_3_)_4_ (42 mg, 0.04 mmol) and CuI (7 mg, 0.04 mmol) were dissolved in dry DMF (7 mL). The mixture was degassed and stirred for 48 h at rt. The solvent was removed under reduced pressure and the residue was dried in vacuo and purified by flash chromatography on silica gel (CH_2_Cl_2_–MeOH 10:1) yielding 27 mg **5** (23%) as a white solid. *R*_f_ 0.53 (CH_2_Cl_2_–MeOH 5:1); ^1^H NMR (300 MHz, CDCl_3_) δ 8.01 (s, 1H, NH), 7.82 (s, 1H, C=CH), 7.70 (s, 2H, H-Ar), 7.55 (d, *J* = 7.8 Hz, 2H, H-Ar), 7.36 (s, 2H, H-Ar), 6.22 (t, *J* = 6.0 Hz, 1H, 1’-H), 4.62 (s, 1H, 3’-H), 4.16–4.02 (m, 1H, 4’-H), 4.00–3.85 (m, 2H, 5’-H), 3.08 (s, 1H, C≡CH), 3.02–2.72 (m, 4H, CH_2_), 2.40 (s, 1H, 2’-H), 2.26 (s, 2H, CH_2_), 2.05–1.79 (m, 7H, CH_3_, 2’-H); ^13^C NMR (75 MHz, CDCl_3_) δ 154.2, 142.7, 141.4, 140.1, 138.9, 138.6, 137.2, 130.0, 127.1, 127.0, 121.6, 118.0, 106.1, 90.4, 88.1, 77.3, 72.8, 64.0, 42.5, 38.5, 24.9, 15.0; MALDI–MS *m*/*z* (%): 633.6 (5) [M]^+^; HRMS–ESI (*m*/*z*): [M + H]^+^ calcd for C_36_H_31_N_2_O_5_S_2_, 635.1674; found, 635.1681.

**Compound 6: 15** (81 mg, 0.20 mmol), 5-iodo-2’-deoxyuridine (**14**, 140 mg, 0.40 mmol), dry NEt_3_ (30 µL, 0.40 mmol), Pd(PPh_3_)_4_ (46 mg, 0.04 mmol) and CuI (8 mg, 0.04 mmol) were dissolved in dry DMF (7 mL). The mixture was degassed and stirred for 3 h at 60 °C. After cooling to rt, the solvent was removed under reduced pressure, and the residue was dried in vacuo and purified by flash chromatography on silica gel (CH_2_Cl_2_–MeOH 15:1) to yield 60 mg **6** (35%) as a white solid. *R*_f_ 0.55 (CH_2_Cl_2_–MeOH 10:1); ^1^H NMR (400 MHz, DMSO-*d*_6_) δ 8.87 (s, 1H, NH), 8.38 (s, 1H, NH), 7.97 (s, 2H, C=CH), 7.85 (d, *J* = 9.3 Hz, 2H, H-Ar), 7.80–7.52 (m, 4H, H-Ar), 6.31–6.02 (m, 2H, 1’-H), 5.31 (d, *J* = 10.7 Hz, 2H, 3’-H), 5.24–5.08 (m, 2H, 4’-H), 4.27 (s, 2H, 5’-H), 3.94–3.83 (m, 2H, 5’-H), 3.79–3.43 (m, 4H, CH_2_), 3.17–3.13 (m, 2H, 2’-H), 3.00–2.67 (m, 10H, 2’-H, CH_2_, CH_3_); ^13^C NMR (101 MHz, DMSO-*d*_6_) δ 161.4, 153.9, 149.4, 137.5, 136.2, 130.3, 130.0, 129.6, 122.8, 120.1, 117.9, 117.6, 106.9, 99.5, 99.2, 98.3, 88.2, 87.6, 84.8, 70.1, 69.5, 60.9, 60.7, 45.6, 37.5, 29.0, 28.7, 23.4, 22.1, 15.0. MS–FAB *m*/*z* (%): 883.4 (25) [M + Na]^+^; HRMS–ESI (*m*/*z*): [M + H]^+^ calcd for C_45_H_41_N_4_O_10_S_2_, 861.2264; found, 861.2282.

**Figure 3:**
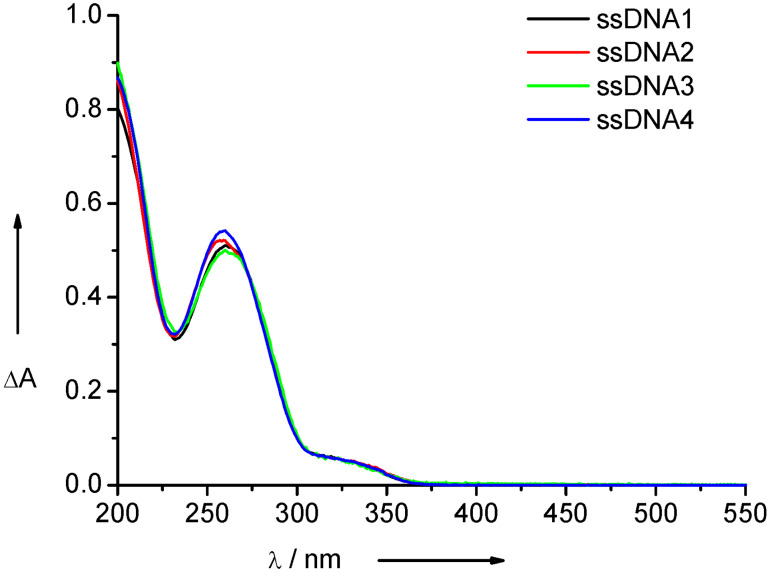
UV–vis absorption spectra of ss**DNA1**–ss**DNA4** (2.5 μM in 50 mM Na–Pi buffer, pH 7, 250 mM NaCl, rt).

**DNA synthesis:** Oligonucleotides were prepared with an Expedite 8909 Synthesizer from ABI by using standard phosphoramidite chemistry. Reagents and CPGs were purchased from ABi and Glen research. Modified oligonucleotides were synthesized by a modified protocol. The activator solution (0.45 M tetrazole in MeCN) was pumped simultaneously with the building block **17** [30] and the coupling time was extended to 35 min, with an intervening step after 17.5 min for washing and refreshing of the activator–**17** solution. After coupling, the vial was washed with MeCN. When the synthesis was complete the trityl-on oligonucleotides were treated with conc. NH_4_OH (700 μL, >25%, trace select, Fluka) at 55 °C for 14 h for cleavage from the resin for deprotection. The oligonucleotides were purified by HPLC on a semipreparative RP-18 column (300 Å, Supelco) by using NH_4_OAc buffer (pH = 6.5) and MeCN as eluents (0–20% MeCN over 70 min; flow rate: 2.5 mL; UV detection: 260 nm, 310 nm). To cleave the terminal DMT group the oligonucleotides were treated with 80% acetic acid for 1 h at rt. After removal of the solvent, the residue was redissolved in H_2_O and the emerging precipitate was removed. The oligonucleotides were lyophilized and quantified by their absorbance in H_2_O at 260 nm on a Varian Cary 100 spectrometer, including ε_260_ = 56200 M^−1^ cm^−1^ for modification **S** (Scheme 3). ds**DNA1** was formed by heating to 90 °C (10 min) followed by slow cooling to rt. MALDI–MS **DNA1** *m*/*z* (%): 5898.9 (100) [M + DMT]^−^, 5597.3 (99) [M]^−^, 2949.2 (21) [M + DMT]^−^/2, 2797.7 (20) [M]^2−^/2; **DNA2 ***m*/*z* (%): 5950.0 (100) [M + DMT]^−^, 5647.9 (38) [M]^−^, 2974.6 (17) [M + DMT]^2−^/2, 2823.6 (7) [M]^2−^/2; **DNA3 ***m*/*z* (%): 5869.0 (100) [M + DMT]^−^, 5666.8 (58) [M]^−^, 2944.4 (12) [M + DMT]^2−^/2, 2782.6 (12) [M]^2−^/2; **DNA4 ***m*/*z* (%): 5919.9 (100) [M + DMT]^−^, 5617.7 (34) [M]^−^, 2957.7 (25) [M + DMT]^2−^/2, 2808.9 (6) [M]^2−^/2. The UV–vis absorption spectra of ss**DNA1**–ss**DNA4** are shown in Figure 3.

## References

[1] Malinovskii V L, Wenger D, Häner R (2010). Chem Soc Rev.

[2] Ruiz-Carretero A, Janssen P G A, Kaeser A, Schenning A P H J (2011). Chem Commun.

[3] Bandy T J, Brewer A, Burns J R, Marth G, Nguyen T, Stulz E (2011). Chem Soc Rev.

[4] Varghese R, Wagenknecht H-A (2009). Chem Commun.

[5] Raymo F M, Tomasulo M (2006). Chem–Eur J.

[6] Cusido J, Deniz E, Raymo F M (2009). Eur J Org Chem.

[7] Pischel U (2007). Angew Chem, Int Ed.

[8] Szaciłowski K (2008). Chem Rev.

[9] Renner C, Moroder L (2006). ChemBioChem.

[10] Berkovic G, Krongauz V, Weiss V (2000). Chem Rev.

[11] Irie M (2000). Chem Rev.

[12] Mayer G, Heckel A (2006). Angew Chem, Int Ed.

[13] Fehrentz T, Schönberger M, Trauner D (2011). Angew Chem, Int Ed.

[14] Asanuma H, Liang X, Yoshida T, Komiyama M (2001). ChemBioChem.

[15] Nishioka H, Liang X, Kashida H, Asanuma H (2007). Chem Commun.

[16] Liang X, Takenaka N, Nishioka H, Asanuma H (2008). Chem–Asian J.

[17] Nishioka H, Liang X, Asanuma H (2010). Chem–Eur J.

[18] Yamazawa A, Liang X, Asanuma H, Komiyama M (2000). Angew Chem, Int Ed.

[19] Liang X, Asanuma H, Komiyama M (2002). J Am Chem Soc.

[20] Liang X, Nishioka H, Takenaka N, Asanuma H (2008). ChemBioChem.

[21] Nishioka H, Liang X, Kato T, Asanuma H (2012). Angew Chem, Int Ed.

[22] Sakata T, Yan Y, Marriott G (2005). J Org Chem.

[23] Young D D, Deiters A (2008). ChemBioChem.

[24] Andersson J, Li S, Lincoln P, Andréasson J (2008). J Am Chem Soc.

[25] Hammarson M, Andersson J, Li S, Lincoln P, Andréasson J (2010). Chem Commun.

[26] Asanuma H, Shirasuka K, Yoshida T, Takarada T, Liang X, Komiyama M (2001). Chem Lett.

[27] Zhang P, Meng J B, Matsuura T, Wang Y M (2002). Chin Chem Lett.

[28] Beyer C, Wagenknecht H-A (2010). J Org Chem.

[29] Beyer C, Wagenknecht H-A (2010). Synlett.

[30] Barrois S, Beyer C, Wagenknecht H-A (2012). Synlett.

[31] Stafforst T, Hilvert D (2009). Chem Commun.

[32] Vomasta D, Högner C, Branda N R, König B (2008). Angew Chem, Int Ed.

[33] Mammana A, Carroll G T, Areephong J, Feringa B L (2011). J Phys Chem B.

[34] Singer M, Jäschke A (2010). J Am Chem Soc.

[35] Ranasinghe R T, Brown T (2005). Chem Commun.

[36] Waggoner A (2006). Curr Opin Chem Biol.

[37] Cobb A J A (2007). Org Biomol Chem.

[38] Wang Y, Haze O, Dinnocenzo J P, Farid S, Farid R S, Gould I R (2007). J Org Chem.

[39] Raytchev M, Mayer E, Amann N, Wagenknecht H-A, Fiebig T (2004). ChemPhysChem.

[40] Sinkeldam R W, Greco N J, Tor Y (2010). Chem Rev.

[41] Sindbert S, Kalinin S, Nguyen H, Kienzler A, Clima L, Bannwarth W, Appel B, Müller S, Seidel C A M (2011). J Am Chem Soc.

[42] Kool E T (2002). Annu Rev Biochem.

[43] Henry A A, Romesberg F E (2005). Curr Opin Biotechnol.

[44] Rist M, Amann N, Wagenknecht H-A (2003). Eur J Org Chem.

[45] Wagner C, Rist M, Mayer-Enthart E, Wagenknecht H-A (2005). Org Biomol Chem.

[46] Ehrenschwender T, Wagenknecht H-A (2008). Synthesis.

[47] Varghese R, Wagenknecht H-A (2009). Chem–Eur J.

[48] Varghese R, Wagenknecht H-A (2010). Chem–Eur J.

[49] Wanninger-Weiß C, Di Pasquale F, Ehrenschwender T, Marx A, Wagenknecht H-A (2008). Chem Commun.

[50] Staiger N, Marx A (2010). ChemBioChem.

[51] Sonogashira K (2002). J Organomet Chem.

